# Impact of Prior Healthcare-Associated Exposure on Clinical and Molecular Characterization of Methicillin-Susceptible *Staphylococcus aureus* Bacteremia: Results From a Retrospective Cohort Study

**DOI:** 10.1097/MD.0000000000000474

**Published:** 2015-02-06

**Authors:** Pao-Yu Chen, Yu-Chung Chuang, Jann-Tay Wang, Shan-Chwen Chang

**Affiliations:** From the Department of Internal Medicine, National Taiwan University Hospital, Jin-Shan Branch, New Taipei, Taiwan (P-YC); Department of Internal Medicine, National Taiwan University Hospital, Taipei, Taiwan (Y-CC, J-TW, S-CC); National Institute of Infectious Diseases and Vaccinology, Miaoli County, Taiwan (J-TW); College of Medicine, National Taiwan University, Taipei, Taiwan (S-CC).

## Abstract

By virtue of medical advances and an aging society, people have increased opportunities for healthcare exposure. Little is known about the impact of healthcare exposure on the clinical features and molecular typing of methicillin-susceptible *Staphylococcus aureus* (MSSA) bacteremia. We classified the onset of MSSA bacteremia into 3 mutually exclusive categories according to the Centers for Disease Control definition, and conducted a retrospective cohort study to investigate the differences among patients with community-associated (CA), healthcare-associated community onset (HACO), and hospital onset (HO) MSSA bacteremia at a medical center from January 1, 2002 through December 31, 2011. Antibiotic susceptibilities and multilocus sequence typing of MSSA isolates were also determined.

A total of 290 patients with MSSA bacteremia, including of 165 (56.9%), 91 (31.4%), and 34 (11.7%) of HACO, HO, and CA, respectively, were studied. ST188 (29.3%) was the most common sequence type regardless of classification. Patients with HACO bacteremia were significantly older, had more solid tumors, higher Charlson scores, and more catheter-related bloodstream infections than those with CA bacteremia. The proportions of osteoarticular infections among patients with both HACO and CA bacteremia were higher than that of patients with HO bacteremia. By univariate analysis, patients with HO bacteremia had significantly higher in-hospital mortality compared to those with CA or HACO bacteremia (31.9% vs 18.8% and 20.4%). Multivariate analysis showed that Charlson score (odds ratio [OR], 1.29; 95% confidence interval [CI], 1.10–1.52), septic shock (OR, 5.28; 95% CI, 2.37–11.78), liver cirrhosis (OR, 3.57; 95% CI, 1.14–11.24), receipt of β-lactams other than oxacillin and cefazolin as definitive therapy (OR, 9.27; 95% CI, 4.25–20.23), and higher oxacillin minimum inhibitory concentration (MIC) (≥0.5 mg/L) (OR, 2.35; 95% CI, 1.05–5.25) of the causative pathogen were independently associated with in-hospital mortality.

In conclusion, patients with HACO bacteremia had different host factors compared with those with CA bacteremia. Infection foci varied with different onset settings. Overall, ST188 was the most predominant sequence type. Onset settings were not independently associated with outcomes.

## Abstract 2

Supplemental Digital Content is available in the text

## INTRODUCTION

*Staphylococcus aureus* is a common human pathogen both in the community and in the hospital setting.^1–3^ However, the incidence of methicillin-resistant *S aureus* (MRSA), especially hospital-onset MRSA infection, has reportedly decreased since the late 2000s,^2–4^ whereas some studies showed that those of methicillin-susceptible *S aureus* (MSSA) infection have increased or at least remained stable.^2,5,6^ Among these, a multinational population-based surveillance demonstrated that the overall annual incidence rate of MSSA bacteremia was 10-fold higher than that of MRSA bacteremia from 2000 to 2008.^6^ The incidence of MSSA bacteremia has increased especially among the elderly in the community.^6^ Therefore, MSSA remains responsible for a great burden of disease in the world.

With medical advances and an aging society, people are increasingly exposed to the healthcare environment and invasive devices even when they were not hospitalized. In studies on MRSA infections, exposure to healthcare-associated risk factors was associated with different clinical syndromes, clonal types, and antibiotic resistance patterns.^5,7,8^ Prior healthcare-associated exposure might also play a role in the clinical spectrum of MSSA bacteremia.

Nevertheless, no studies have evaluated whether community-onset MSSA bacteremia with healthcare-associated risks differ from those without risks with respect to clinical features and molecular characterization.^8–13^ In addition, little is known regarding the impact of increasing healthcare exposure on the evolutionary changes in molecular typing of MSSA in the community. This study therefore aimed to compare the clinical features of adult patients with MSSA bacteremia and the longitudinal molecular typing of causative isolates among hospital onset (HO) and community onset with or without healthcare-associated risks.

## MATERIALS AND METHODS

### Study Population

This retrospective cohort study was conducted at the National Taiwan University Hospital (NTUH), a major 2200-bed medical center in Northern Taiwan. Patients with concomitant bloodstream infections by other microorganisms were excluded. One of every 5 patients ≥18 years old with MSSA bacteremia diagnosed between January 1, 2002 and December 31, 2011 was randomly sampled in each year using a computer-generated random digital number table. For patients with multiple episodes of MSSA bacteremia during the study period, only the first episode was included. The study was approved by the Institutional Review Board (IRB) at NTUH (IRB_201303097RINC).

### Microbiological Testing

Blood cultures were processed by the NTUH microbiology laboratory using the Bactec 9240 system (Becton Dickson, Sparks, MD). *S aureus* was primarily identified using biochemical methods and the Phoenix bacterial identification system (Becton Dickson Diagnostic System) as described previously.^5^ All blood isolates of *S aureus* have been prospectively preserved in the research laboratory of the Department of Internal Medicine at NTUH since the early 1990s. Antimicrobial susceptibilities to oxacillin (using cefoxitin disk), gentamicin, clindamycin, minocycline, erythromycin, trimethoprim-sulfamethoxazole, and fusidic acid were determined by the disk diffusion method.^14^ The minimum inhibitory concentrations (MICs) of oxacillin and vancomycin were determined by agar dilution methods. The MICs tests were repeated and the results were interpreted according to Clinical and Laboratory Standards Institute (CLSI) criteria.^15^ Multilocus sequence typing (MLST) was performed as described previously.^16^

### Data Collection

A standardized case report form was used to collect information on the patients’ demographic and clinical data by chart review. The Charlson comorbidity index^17^ was used to evaluate the underlying conditions. The infection focus of bacteremia was diagnosed on the basis of clinical, bacteriological, and radiological results as described previously.^5,18^ The infection was considered “deep-seated” if any of the following were present: infective endocarditis, mycotic aneurysm, osteomyelitis, septic arthritis, pyomyositis, necrotizing pneumonia/empyema, or abscess formation in any deep organ, such as the liver or kidneys. Endovascular infection included infective endocarditis or mycotic aneurysm. If no infection focus could be identified, the bacteremia was classified as primary bacteremia. We classified MSSA bacteremia as HO, healthcare-associated community onset (HACO), or community-associated (CA) using the Centers for Disease Control and Prevention definition for MRSA.^5,7,19,20^ “HO” was defined by an MSSA-positive blood culture obtained more than 48 hours after admission. “HACO” was defined as an index blood culture collected less than 48 hours after admission with the presence of the following risk factors within the past year: (1) residence in a long-term care facility, (2) prior admission to an acute care facility, (3) the use of central intravenous catheters or long-term venous access devices, (4) the use of urinary catheters, (5) the use of other long-term percutaneous devices, (6) prior surgical procedures, and/or (7) the need for any form of dialysis. “CA” was defined as an index blood culture collected within 48 hours of admission without the aforementioned risk factors within the past year. The definitive antibiotic therapy was defined as the effective antibiotic used after the susceptibility test result was available. We classified definitive antibiotic therapy as oxacillin, cefazolin, and effective β-lactams (to which MSSA isolates were susceptible by antimicrobial susceptibility test results) other than oxacillin and cefazolin. Source control was defined as the removal of a medical device or surgical debridement of deep-seated foci as clinically indicated. Laboratory parameters assessed 24 hours before and/or after MSSA bacteremia onset included white blood cell count, hemoglobin, platelet count, C-reactive protein, albumin, creatinine, and liver function (ie, aspartate aminotransferase and total bilirubin). The endpoint was all-cause in-hospital mortality.

### Statistical Analysis

Annual incidence rates of MSSA bacteremia were calculated as the number of cases of MSSA per 1000 discharge. The increasing trend via time of incidence was examined by a logistic regression analysis.^21^ Continuous variables are expressed as medians and interquartile ranges (IQRs), and categorical variables as percentages. The associations between the clinical presentations of MSSA bacteremia among the 3 onset settings were compared using the Kruskal–Wallis one-way analysis of variance (ANOVA) or Fisher's exact test where appropriate. Post hoc analysis using the Mann–Whitney *U*-test or Fisher's exact test with a Bonferroni-adjusted α for pairwise comparisons was performed if the result of the initial test was statistically significant. Risk factors for mortality were identified by logistic regression analysis. All relevant clinical and laboratory variables, as well as the biologically potential interaction terms between these variables were first entered into univariate analysis. Variables with a *P*-value <0.20 and probable biological meaning were subsequently entered into the multivariate analysis. Backwards stepwise model comparison and selection were used to determine the final model of multivariate analysis. All tests were 2-tailed. The level of statistical significance was set at *P* < 0.05. All analyses were performed using SPSS for Windows (Release 18.0; SPSS, Chicago, IL).

## RESULTS

### Patient Characteristics and Infection Focus

The annual incidences of MSSA bacteremia at NTUH remained stable (*P* = 0.99, by logistic regression test for trend), ranging from 1.43 to 1.89 per 1000 discharges over time (Figure S1 http://links.lww.com/MD/A172). A total of 290 adult patients were randomly selected and enrolled in this study. Among them, 165 (56.9%), 91 (31.4%), and 34 (11.7%) patients were classified as HACO, HO, and CA bacteremia, respectively. The clinical characteristics of these patients in these 3 groups are shown in Table 1. Patients with HACO bacteremia were significantly older, had significantly more solid tumors, and higher Charlson scores than patients with CA bacteremia (*P* = 0.001, <0.001, and <0.001, respectively). Patients with HACO and HO bacteremia had similar host and clinical characteristics, except the former group had significantly fewer hematological malignancies (*P* = 0.004). The primary foci for MSSA bacteremia are shown in Table 2. Significantly more patients with HACO and HO bacteremia had a central catheter as their primary focus than those with CA bacteremia (*P* = 0.01 and < 0.001, respectively). Meanwhile, osteoarticular infection was significantly more common as the source of bacteremia in HACO and CA bacteremia patients than HO bacteremia patients (*P* = 0.01 and <0.001, respectively). The proportions of patients with endovascular infection and pyomyositis were both highest in patients with CA bacteremia group (20.0% and 14.3%, respectively.)

**TABLE 1:**
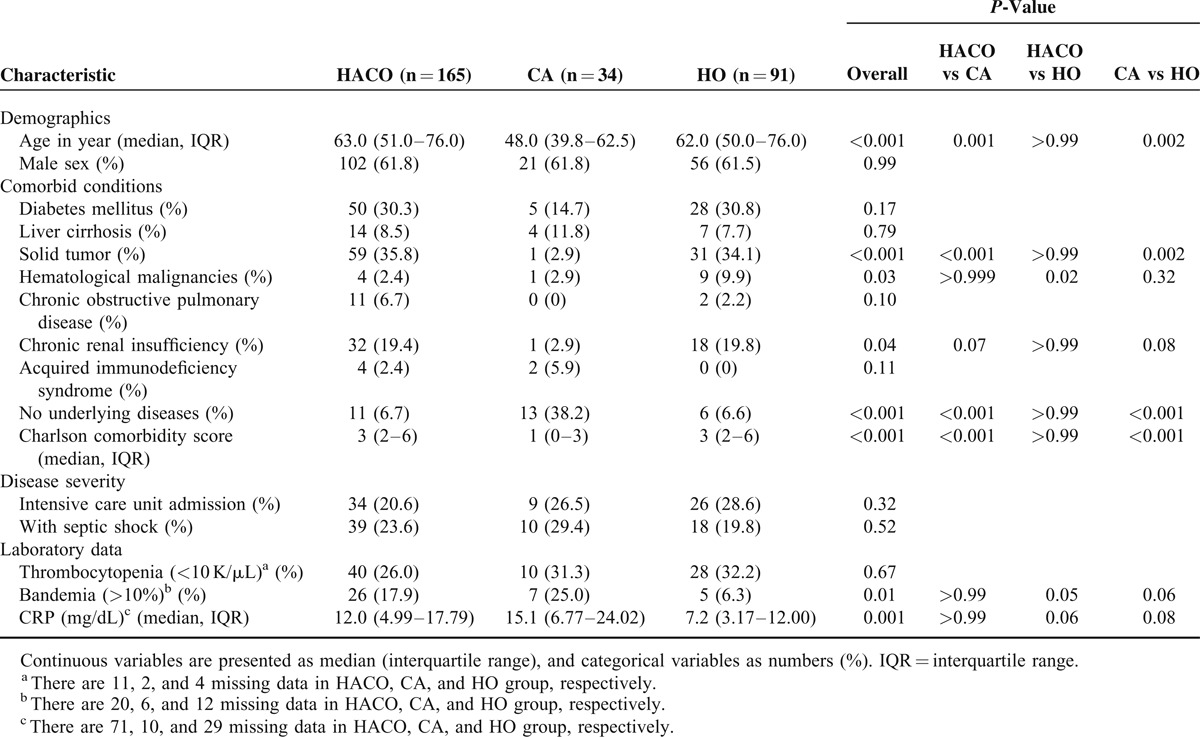
Comparison of Clinical Characteristics of MSSA Bacteremia Patients Classified by Onset as Healthcare-Associated Community Onset (HACO), Community Associated (CA), and Hospital Onset (HO)

**TABLE 2:**
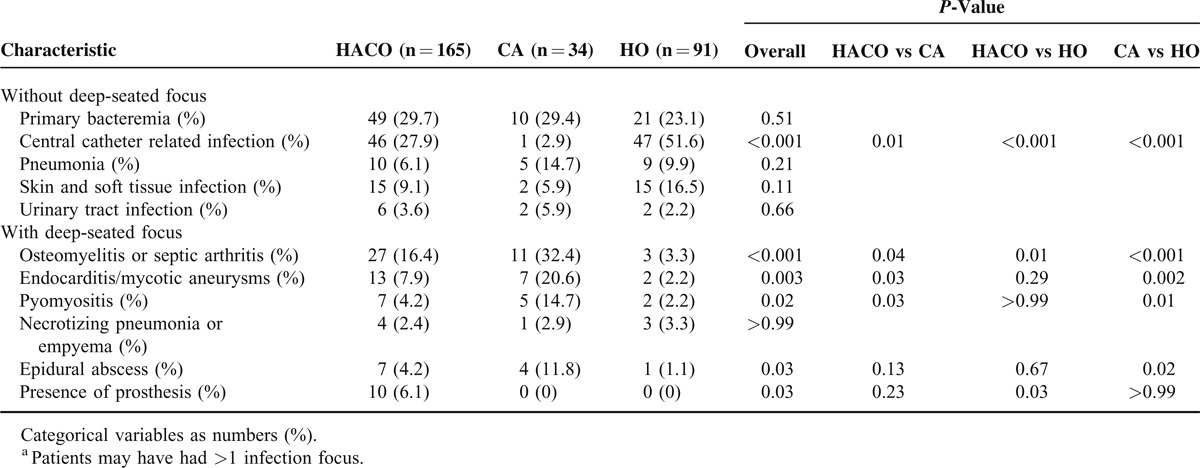
Comparison of Infection Foci^a^ of MSSA Bacteremia Patients Classified as Healthcare-Associated Community Onset (HACO), Community Associated (CA), and Hospital Onset (HO)

### Antimicrobial Susceptibility Test Results

**FIGURE 1:**
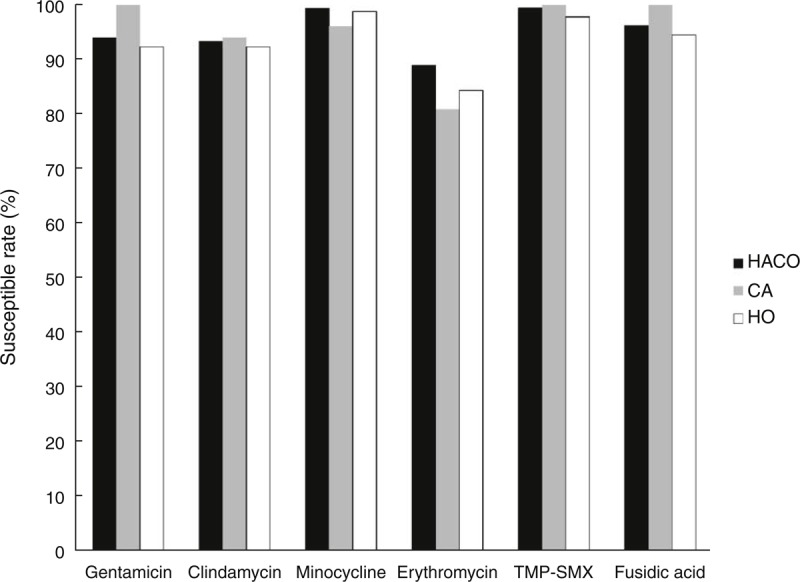
Antibiograms of MSSA blood isolates from 2002 to 2011 stratified according to onset setting. CA = community-associated; HACO = healthcare-associated community onset; HO = hospital onset; TMP-SMX = trimethroprim-sulfamethoxazole.

**FIGURE 2:**
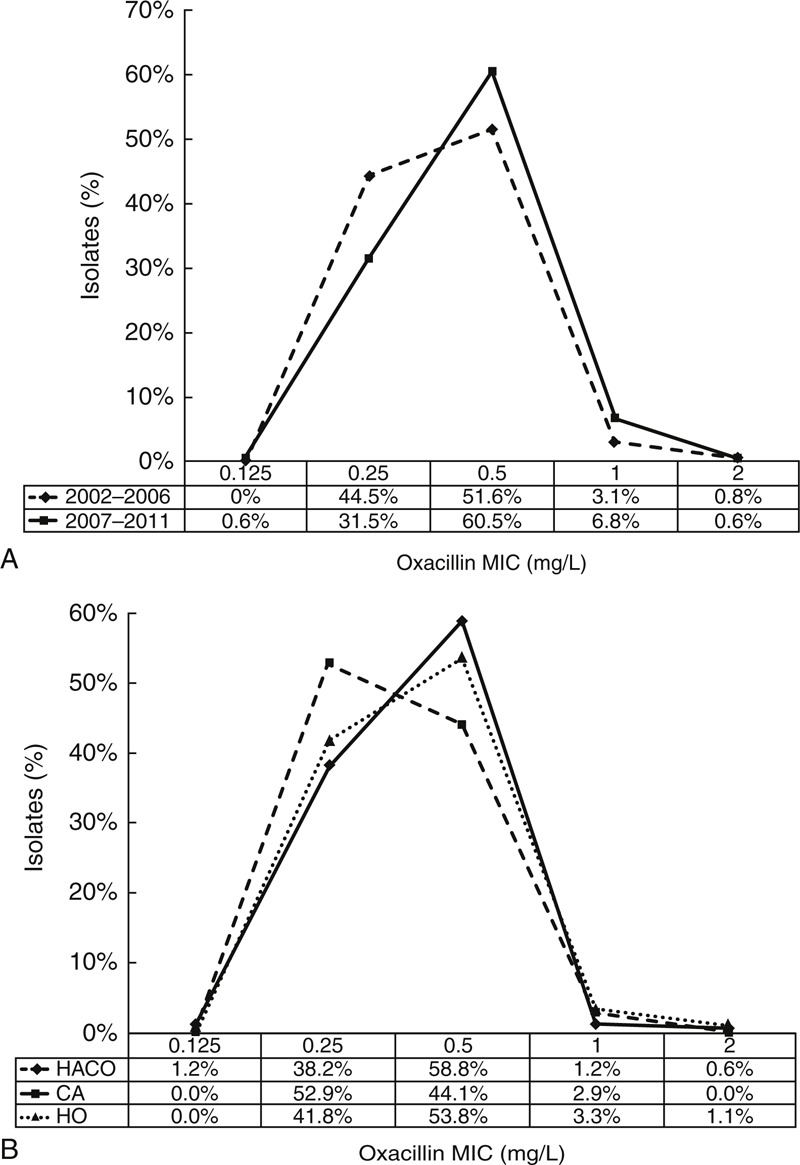
Distribution of oxacillin minimum inhibitory concentrations of MSSA blood isolates from 2002 to 2011 stratified according to study period (A) and onset setting (B). Numbers below the X-axis indicate the percentages of total isolates in each group. CA = community-associated; HACO = healthcare-associated community onset; HO = hospital onset.

All of the 290 bacteremia-related isolates were available for microbiological investigations. The antimicrobial susceptibilities of MSSA blood isolates stratified according to onset settings are shown in Figure 1. The overall susceptibility rates to gentamicin, clindamycin, minocycline, trimethroprim-sulfamethoxazole, and fusidic acid all exceeded 90%, but the overall susceptibility rate to erythromycin was only 86.6%. There were no significant differences in the susceptibilities to tested drugs among the 3 groups. All isolates were susceptible to oxacillin and vancomycin. There was no creeping of oxacillin MIC stratified by the study period (Figure 2A) and onset setting (Figure 2B). Vancomycin MIC also remained stable over time (Figure S2 http://links.lww.com/MD/A173, http://links.lww.com/MD/A174).

### Molecular Typing

**FIGURE 3:**
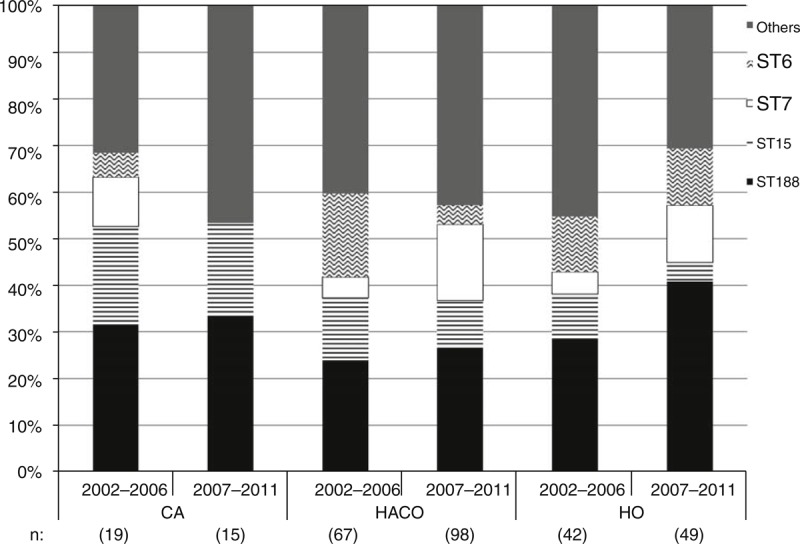
Distribution of sequence types of MSSA blood isolates from 2002 to 2011 stratified according to onset setting and study period. Numbers below the X-axis indicate the numbers of total isolates in each group. CA = community-associated; HACO = healthcare-associated community onset; HO = hospital onset.

Among these 290 isolates, ST188 was the most common sequence type (ST) (29.3%), followed by ST15 (11.0%), ST7 (10.0%), ST6 (9.7%), and ST59 (5.2%). These STs accounted for 65.2% of all isolates. The distribution of MLST is shown in Table S1 http://links.lww.com/MD/A171. The proportions of ST188 causing CA, HACO, and HO MSSA bacteremia were 32.4%, 25.5%, and 35.5%, respectively (*P* = 0.52). ST188 remained the predominant one in both the first and second 5-year periods among all 3 onset settings (Figure 3). Comparing the patients’ characteristics, antimicrobial susceptibilities, and outcomes, there were no significant differences between patients with bacteremia caused by ST188 and other STs (Table S2 http://links.lww.com/MD/A171).

### Management and Outcome Analysis

Regarding the definitive antibiotic therapy, 141 (48.6%), 29 (10.0%), and 115 (39.7%) patients received oxacillin, cefazolin, and β-lactams other than oxacillin and cefazolin, respectively. Only 5 (1.7%) patients received glycopeptide. Patients with CA and HACO bacteremia were more likely to receive oxacillin. On the other hand, patients with HO bacteremia had the highest proportion of receiving β-lactams other than oxacillin and cefazolin (Table 3, *P* = 0.03).

**TABLE 3:**
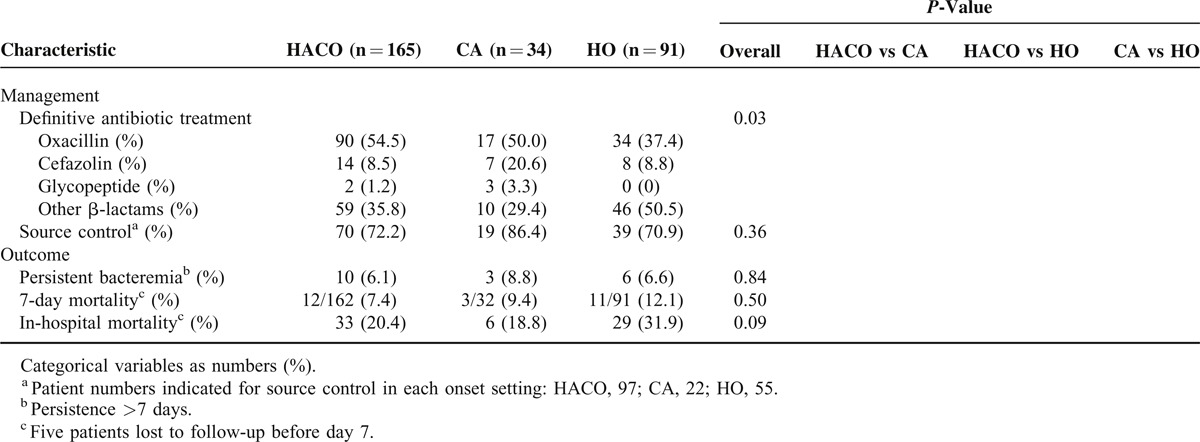
Comparison of Management and Outcome of MSSA Bacteremia Patients Classified by Onset With Different Onset Settings as Healthcare-Associated Community Onset (HACO), Community Associated (CA) and Hospital Onset (HO)

The median follow-up durations of patients with MSSA bacteremia among CA, HACO, and HO settings were 31.0 days (interquatile range [IQR], 14.5–50.5 d), 24.0 days (IQR, 13.5–34.0 d), and 16.0 days (IQR, 11.0–42.0 d), respectively. A total of 285 patients with MSSA bacteremia were included in the outcome analysis after excluding 5 patients who were lost to follow-up before day 7. The all-cause in-hospital mortality rate was 23.4%. Patients with HO bacteremia had the highest rate (31.9%), while patients with CA or HACO bacteremia had similar rates (18.8% and 20.4%, respectively). The comparison of in-hospital mortality between the 3 onset settings revealed a borderline significant difference (*P* = 0.09).

Logistic regression analysis of the significant predictors of mortality is shown in Table 4. In univariate analysis, the presence of solid tumors, Charlson score, intensive care unit (ICU) admission, septic shock, HO bacteremia, pneumonia, deep-seated infection, thrombocytopenia, bandemia, and receipt of effective β-lactams other than oxacillin and cefazolin as definitive therapy were associated with in-hospital mortality. By multivariate logistic regression analysis, Charlson score (odds ratio [OR], 1.29; 95% confidence interval [CI], 1.10–1.52; *P* = 0.002), septic shock (OR, 5.28; 95% CI, 2.37–11.78; *P* < 0.001), liver cirrhosis (OR, 3.57; 95% CI, 1.14–11.24; *P* = 0.03), receipt of β-lactams other than oxacillin and cefazolin (OR, 9.27; 95% CI, 4.25–20.23; *P* < 0.001) and higher oxacillin MIC (≥0.5 mg/L) (OR, 2.35; 95% CI, 1.05–5.25; *P* = 0.04) of the causative pathogen were independent predictors of in-hospital mortality. Different onset settings, and sequence type of causative MSSA were not associated with in-hospital mortality.

**TABLE 4:**
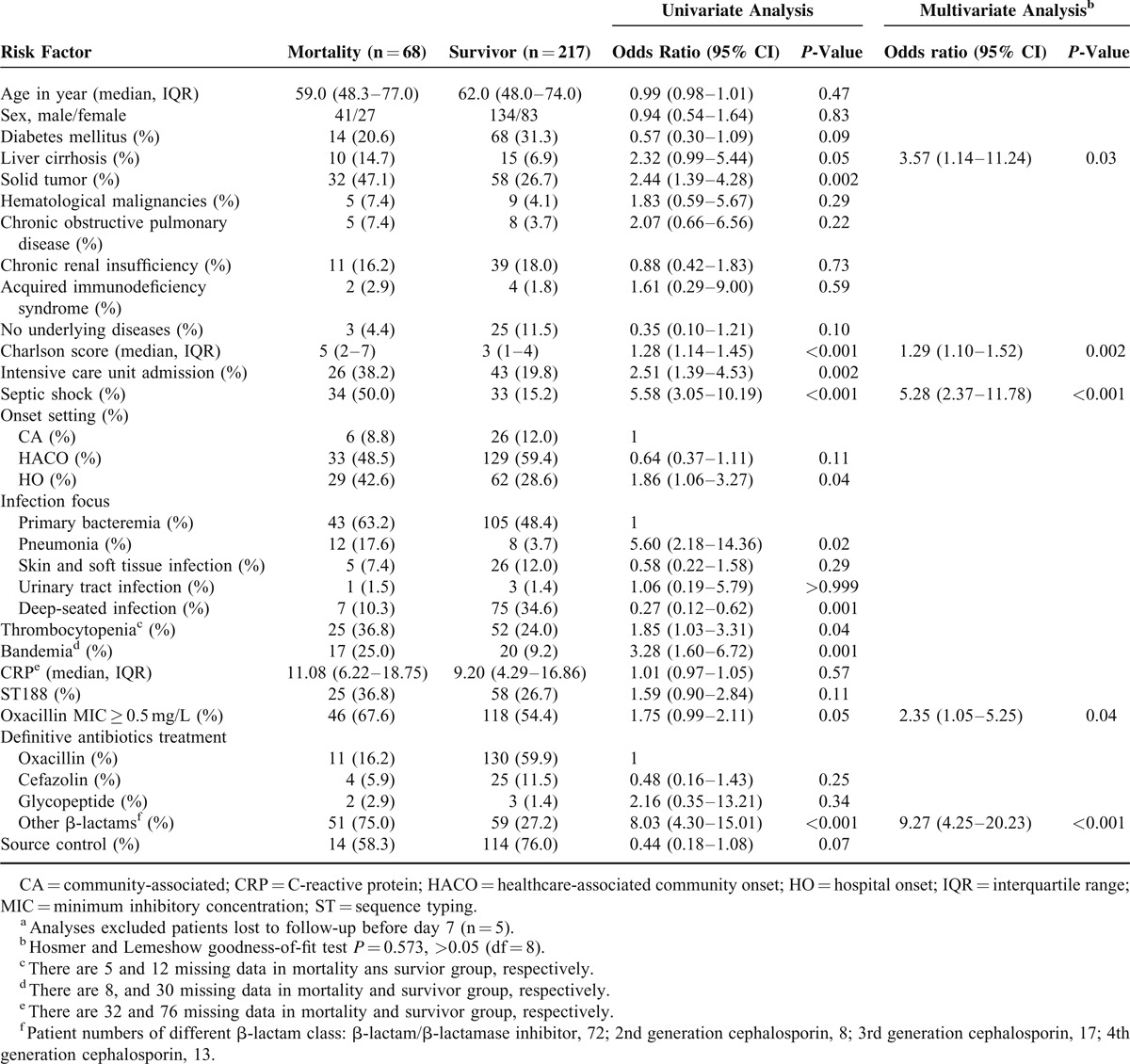
Logistic Regression Analysis of Predictors of In-Hospital Mortality in Patients^a^ With MSSA Bacteremia

## DISCUSSION

Although a previous study demonstrated that community onset MSSA bacteremia had different clinical features from HO bacteremia,^12^ there was no discussion on whether prior healthcare-associated exposure affected the demographic or clinical presentations of patients with MSSA bacteremia of community onset. The present results show that patients with MSSA bacteremia in HACO settings had unique disease characteristics compared to those in CA or HO settings. The host factors of patients with MSSA bacteremia in HACO settings were similar to those in HO settings. On the other hand, the distribution of primary foci of MSSA bacteremia in HACO settings constituted a mixture of those in CA and HO settings. Also, our study demonstrated a longitudinal distribution of molecular typing of MSSA blood isolates.

In the present study, patients with HACO and HO bacteremia were significantly older and had more underlying diseases than those with CA bacteremia, which was in agreement with previous studies regarding all bloodstream infections.^22^ These findings are reasonable because elderly patients or patients with underlying diseases require more medical care than young or healthy adults; hence contributing to their higher probability of exposure to health care. Our study further compared the infection foci of MSSA bacteremia among different onset settings. The proportions of osteoarticular infections among patients with both HACO and CA bacteremia were higher than that of patients with HO bacteremia. Apart from this, patients with CA setting had the highest proportions of pyomyositis and endovascular infections as their primary foci. Clinicians may thus prioritize surveillance of certain occult primary foci according to the setting of MSSA bacteremia. The possible underlying mechanisms to explain this phenomenon include different host characteristics, microbiological features^23^ or exposure history among the 3 onset settings, akin to that demonstrated for MRSA infection.^24^ However, the present results suggest that the role of microbiological differences may be less important than host factors, since the distributions of molecular typing were similar among these patient groups.

Our study is concordant with previous studies in demonstrating the genetic diversity of MSSA causing clinical infectious syndromes.^3,9,10,13,25–30^ However, ST188 was identified as the major strain of MSSA from blood isolates during the 10-year study period. The records of the Infection Control Center at NTUH during the study period indicate there was no outbreak of MSSA bacteremia in our hospital. Recent studies reported that ST188 was a common strain in Asia.^27–30^ Our study echoed this phenomenon and showed additional information that ST188 was consistently prevalent in CA, HACO, and HO infections in Taiwan from 2002 to 2011. Unlike MRSA,^24^ the present study did not show different distribution of molecular typing among CA, HACO and HO settings. This finding is interesting and its underlying mechanisms need further investigation. Of note, only 1 isolate belonged to ST398, which has emerged in the USA and Europe.^31,32^ This suggests that the molecular characterization of MSSA also exhibits geographical differences like MRSA strains.

Traditional risk factors such as comorbidities and septic shock have been associated with mortality from MSSA bacteremia in this and other studies.^5,12,19^ The present study revealed 2 other predictors of mortality from MSSA bacteremia. The first is the receipt of β-lactams other than oxacillin and cefazolin as definitive therapy for MSSA bacteremia. This result should be interpreted conservatively because patients receiving β-lactams other than oxacillin and cefazolin as definitive therapy were more likely to be in the HO setting, and patients in HO setting had more comorbidities (Table 1). Besides, the dosages and serum concentrations of used antibiotics were not available for further analysis due to our retrospective design.

The other is that a high oxacillin MIC (≥0.5 mg/L) was significantly associated with increased in-hospital mortality. Our finding is biologically plausible since a higher oxacillin MIC would result in a shorter time interval when the serum concentration was above MIC, which in turn might compromise the effectiveness of oxacillin and other time-dependent beta-lactams.^33,34^ In contrast to 2 reports showing a higher vancomycin MIC associated with increased mortality in patients with MSSA bacteremia with high proportions of MSSA isolates with vancomycin MIC > 1 mg/L by E-test, only 1 of our MSSA isolates (0.3%) had vancomycin MIC >1 mg/L by agar dilution in our study.^35,36^ Therefore, we were unable to assess the association between mortality and vancomycin MIC. These 2 aforementioned predictors for mortality of MSSA bacteremia are interesting, but dedicated analyses are beyond the scope of the current study. Therefore, further specifically designed studies were warranted to investigate the impact of different β-lactams and oxacillin MIC on outcomes of patients with MSSA bacteremia.

This study has several limitations. First, this was a retrospective single-center study. Therefore, missing data and potential information bias is inevitable, and caution should be taken when generalizing the results to other institutions. Second, only blood isolates were analyzed. Thus, the molecular epidemiological findings may not be applicable to other invasive MSSA infections. Third, only one-fifth of patients with MSSA bacteremia were sampled because the total population of MSSA bacteremic patients during 2002 to 2011 was too large to be analyzed. Therefore, sampling bias might be present. However, we minimized this bias through random sampling using a computer-generated random digital number table. Furthermore, the number of patients with CA MSSA bacteremia is relatively small in the present study, which was probably because our hospital is a referral center and many of our patients had prior exposures to healthcare-associated risk factors. This might compromise the statistical power. However, we enrolled relatively larger numbers of patients with HACO or HO MSSA bacteremia (165 and 91, respectively, vs 34), which would partially improve the compromised statistical power.^37^

In conclusion, the present study revealed that patients with HACO MSSA bacteremia differed significantly from those with CA bacteremia with respect to the demographic characteristics, comorbidities, and infection foci. ST188 was the major strain among all 3 onset settings. The in-hospital mortality rate of patients with HACO MSSA bacteremia was similar to that of patients with CA bacteremia but less than that of patients with HO bacteremia by univariate analysis. By multivariate analysis, onset settings did not independently influence mortality. Instead, the Charlson score, liver cirrhosis, septic shock, receipt of β-lactams other than oxacillin and cefazolin, and higher oxacillin MIC were associated with a poorer outcome.
